# Radiomics-Based prediction of renal prognosis in ANCA-associated vasculitis

**DOI:** 10.3389/fdgth.2026.1915391

**Published:** 2026-09-30

**Authors:** Zuoxian Hou, Shuo Zhang, Peng Xia, Shilu Cao, Linkun Bai, Wenling Ye, Feng Feng, Limeng Chen

**Affiliations:** 1 Department of Nephrology, Peking Union Medical College Hospital, Chinese Academy of Medical Sciences, Beijing, China; 2 Beijing Key Laboratory of Molecular Pathology Diagnosis and Translational Research on Rare and Complex Urinary System Diseases, Peking Union Medical College Hospital, Beijing, China; 3 Center for Digital Medicine and Artificial Intelligence, National Infrastructures for Translational Medicine, Peking Union Medical College Hospital, Peking Union Medical College & Chinese Academy of Medical Science, Beijing, China; 4 Department of Radiology, Peking Union Medical College Hospital, Chinese Academy of Medical Sciences, Beijing, China

**Keywords:** ANCA-associated vasculitis (AAV), clinical markers, prediction model, radiomics, renal prognosis

## Abstract

**Objective:**

To develop and validate a non-invasive prediction model combining renal CT radiomics features and clinical markers for assessing long-term renal prognosis at 1, 3, and 5 years in patients with antineutrophil cytoplasmic antibody (ANCA)-associated vasculitis (AAV).

**Methods:**

A retrospective cohort of 262 AAV patients from Peking Union Medical College Hospital (2015–2020) was analyzed. Radiomics features (morphological, first-order, and texture) were extracted from segmented renal parenchyma on abdominopelvic CT. Clinical markers included demographics, serum creatinine, complement component 3 (C3), and other laboratory indicators. Renal volume, serum creatinine, and complement component 3 (C3) were pre-specified as predictors based on *a priori* biological rationale, and XGBoost was employed for model construction. Performance was evaluated via ROC-AUC (receiver operating characteristic/area under the curve), F1 score, decision curve analysis, and calibration curves. SHAP (SHapley Additive exPlanations) analysis assessed feature contributions, and Kaplan–Meier curves with Bonferroni-corrected log-rank tests were used for survival stratification.

**Results:**

The mean follow-up was 40.8 months; 59 patients (23%) developed ESRD (56 within 3 years) and 29 (11%) died. The combined model (renal volume + sCr + C3) achieved an AUC of 0.93 for 3-year ESRD prediction (clinical-only model: 0.91), with renal volume independently associated with ESRD (Wald *p* = 0.004). For the 3-year composite outcome, the combined model achieved an AUC of 0.84 (clinical-only: 0.81) with significantly improved reclassification (IDI *p* = 0.018; category-free NRI *p* = 0.024). In pre-specified median-based Kaplan–Meier analyses, reduced renal volume (≤ 130.6 mL), elevated serum creatinine (> 177.5 μmol/L) and lower C3 (≤ 0.97 g/L) were associated with poorer 3-year ESRD-free survival (log-rank *p* ≤ 0.002).

**Conclusion:**

The integrated radiomics-clinical model provides accurate, non-invasive risk stratification for long-term renal outcomes in AAV, facilitating personalized treatment.

## 1 Introduction

Accurate prediction of renal outcomes is crucial for optimizing treatment intensity and improving survival in patients with antineutrophil cytoplasmic antibody (ANCA)-associated vasculitis (AAV). AAV is a group of systemic autoimmune diseases characterized by necrotizing inflammation of small blood vessels, divided into three main subtypes: microscopic polyangiitis (MPA), granulomatosis with polyangiitis (GPA), and eosinophilic granulomatosis with polyangiitis (EGPA) (1, 2). The kidney is the most commonly involved organ and has the greatest impact on prognosis. It is reported that approximately 38% to 70% of GPA patients and 80% to 100% of MPA patients experience renal involvement. Renal injury caused by AAV is a significant cause of acute kidney injury (AKI) and end-stage renal disease (ESRD), usually presenting as rapidly progressive glomerulonephritis, which is characterized by a rapid decline in renal function, proteinuria, microscopic hematuria, and hypertension over days or weeks (2, 3). Some patients, however, exhibit an insidious progression with a slowly advancing clinical course (4). This marked heterogeneity in disease progression and outcomes poses considerable challenges in clinical decision-making.

Accurate risk stratification helps identify high-risk patients and facilitates personalized treatment planning. At present, various classification scoring systems and prognostic models (5–8) are primarily based on renal histopathology, the proportions of glomerular lesions (such as sclerotic, crescentic, or normal glomeruli), the degree of tubulointerstitial damage (including interstitial fibrosis and tubular atrophy), and baseline renal function (sCr or eGFR). However, these approaches rely on invasive renal biopsy with risks of bleeding and infection and exclude patients with contraindications, thereby limiting their broad applicability in aged patients (9), who are common in patients with AAV. Moreover, these systems offer a limited perspective, either visualizing microscopic lesions in pathology or indirectly estimating renal function without capturing macroscopic structural changes in the kidneys. The images are non-invasive and can be repeatedly obtained. Thus, there is a compelling need to develop a more comprehensive and non-invasive prognostic tool for AAV.

Radiomics, as an emerging discipline that integrates medical imaging and bioinformatics, has its core advantage lies in mining and extracting massive, high-dimensional quantitative features from conventional medical images. These features capture microstructure information and indirectly reflect the subtle changes in their functional status, providing a new technical path for the precise assessment of diseases. In nephrology, radiomics has shown great promise. At present, several studies have confirmed that it can be effectively applied to the staging assessment of chronic kidney disease, the prediction of progression risk of diabetic nephropathy, and other scenarios by quantifying the structural heterogeneity and perfusion of renal tissue, providing an essential auxiliary basis for the clinical management of these common renal diseases.

This study, to our knowledge, is the first to integrate renal radiomics features from CT scans and clinical markers to develop a new strategy for predicting 1-, 3-, and 5-year prognosis, including ESRD and renal composite endpoints, in patients with AAV. The specific objectives of this study were to: (1) develop and validate prediction models for ESRD and composite renal endpoints, with the 3-year endpoint designated as primary and 1-/5-year analyses as exploratory; (2) identify the optimal combination of clinical markers and radiomics features; (3) assess feature contribution using SHAP value analysis; and (4) perform survival analysis to clarify the influence of key indicators on patient prognosis.

## 2 Methods

### 2.1 Clinical data at baseline and outcome variables

#### 2.1.1 Inclusion and exclusion criteria

This study was a retrospective cohort study, enrolling AAV patients hospitalized within a specific period (Figure 1). Inclusion criteria were as follows: (1) Diagnosed as ANCA-associated vasculitis according to the 2012 revised International Chapel Hill Consensus Conference (10); (2) Completed abdominopelvic CT examination within 2 weeks from the hospitalization date; (3) Complete clinical data (demographics, laboratory indicators, treatment plan) and follow-up data (ESRD, death events and corresponding time). Exclusion criteria included: (1) Renal CT image quality was confirmed to be poor by radiologists; (2) Renal region was missing in renal CT images; (3) Complicated with diabetic nephropathy, chronic glomerulonephritis and other diseases that may independently affect renal prognosis. From an initial cohort of 1,203 patients with complete clinical data, 931 did not undergo CT and were not part of the imaging cohort; among the 272 patients who underwent CT, 1 was excluded because the kidney was not fully imaged and 9 because severe image artefacts precluded reliable segmentation (11–14), leaving 262 patients for analysis (Figure 2). This study was approved by the Ethical Committee of Peking Union Medical College Hospital (No. K8557), the requirement for written informed consent was waived because this was a retrospective study.

**Figure 1:**
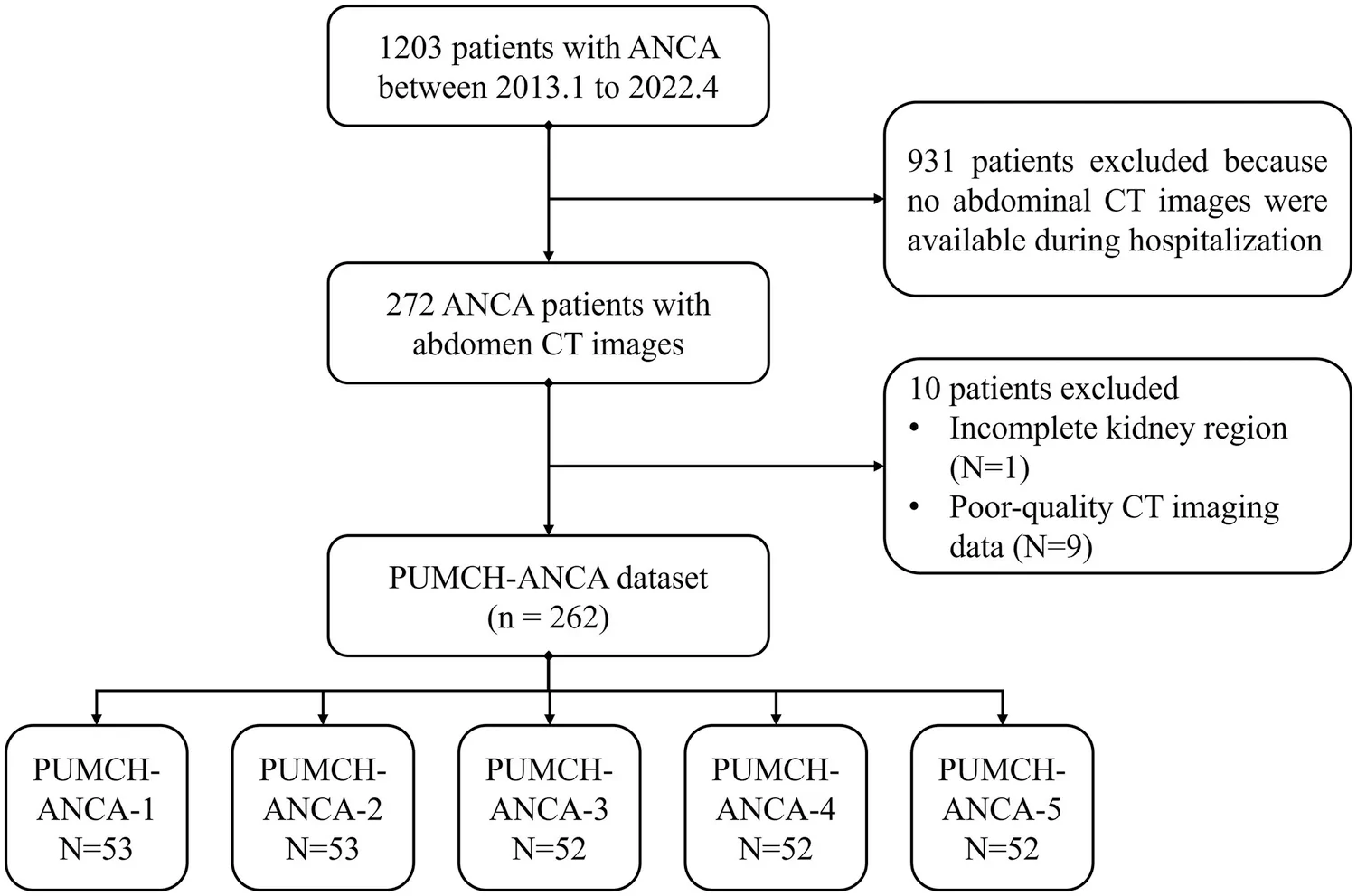
Patient flow of ANCA patients in this study.

**Figure 2:**
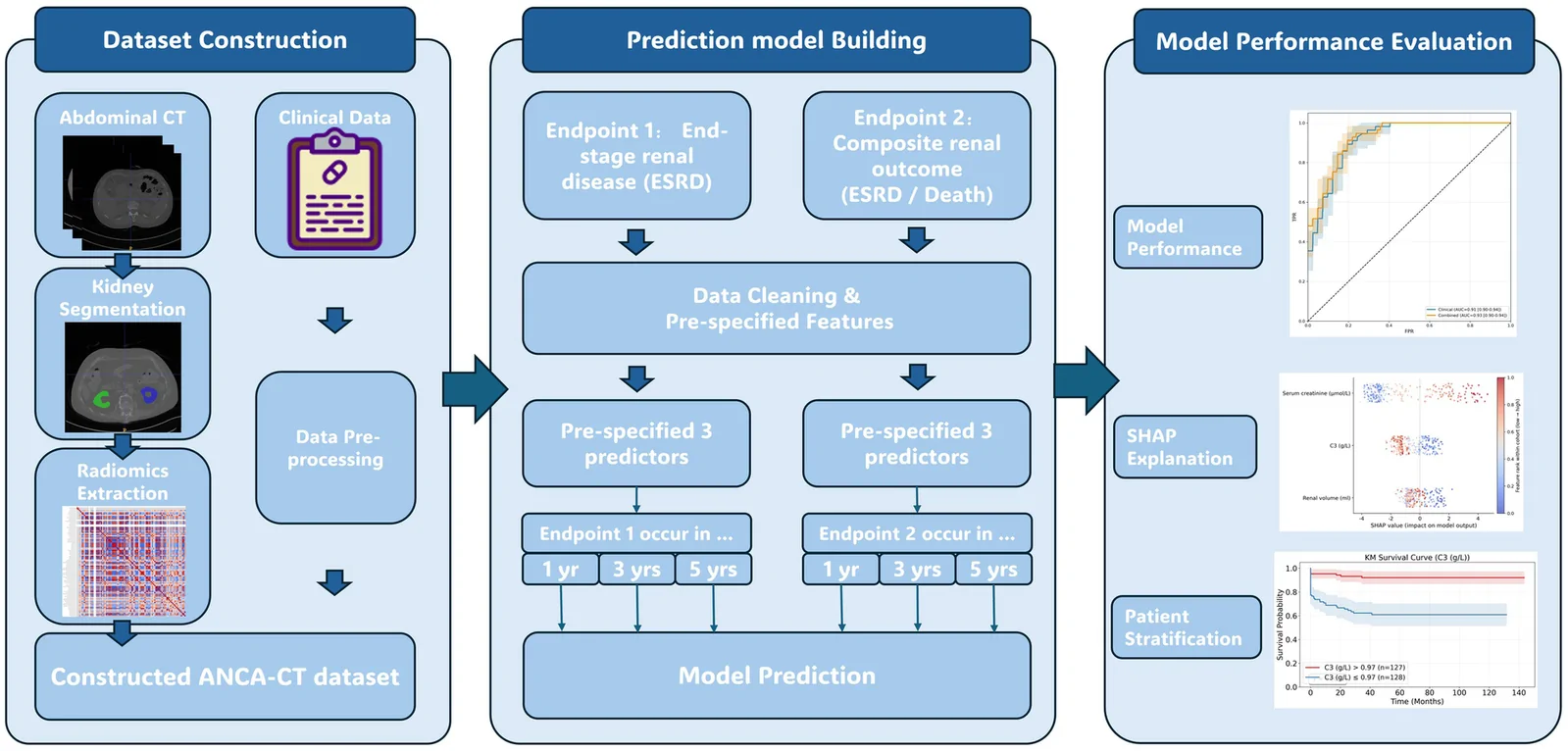
Study design. Dataset construction: our data comprised two parts - patients' clinical data and abdominal CT scans obtained during their first admission due to ANCA-associated vasculitis; the kidney region was segmented, and radiomic features were extracted from the region of interest (ROI); the clinical data were also pre-processed and integrated with the CT radiomics to form the final ANCA-CT dataset. Prediction model building: there were 2 endpoints in this study - end-stage renal disease (ESRD) and composite renal outcome (ESRD/death); the ANCA-CT dataset underwent data cleaning with a pre-specified feature set (renal volume, sCr, C3), and different features were utilized for prediction. Models were also built for reaching the corresponding endpoints at different follow-up time points (1/3/5 years). Model performance evaluation: we used ROC-AUC curves, radar plots, decision curve analysis (DCA) and calibration curves to evaluate model performance; we also employed SHAP (SHapley Additive exPlanations) to explore the importance of each feature, and finally stratified patients into groups to compare the survival curves.

### 2.2 Data collection

Clinical data of patients at baseline (around the time of CT examination) and follow-up data were collected, including age, gender, AAV subtype (MPA, GPA, EGPA), ANCA type (myeloperoxidase MPO-ANCA/proteinase 3 PR3-ANCA), unclassified AAV, past medical history; regarding laboratory indicators, core indicators include serum creatinine (sCr), estimated glomerular filtration rate (eGFR calculated by the CKD-EPI equation), 24-hour urinary protein, and complement component 3 (C3),etc.

All CT examinations were performed without intravenous contrast. Images were acquired on scanners from three vendors: Siemens (SOMATOM Definition Flash, Sensation 64, SOMATOM Definition, SOMATOM go. All, SOMATOM Force; *n* = 213), Philips (Incisive CT, iCT 256, and others; *n* = 34), and GE (Discovery CT750 HD; *n* = 15). Tube voltage was predominantly 120 kV (*n* = 208), with 140 kV (*n* = 35), 100 kV (*n* = 15), 110 kV (*n* = 3), and 90 kV (*n* = 1) in the remainder; the dominant reconstruction kernel was B30f (*n* = 168), with B25f (*n* = 18), B31f (*n* = 15), STANDARD/GE (*n* = 15), and other kernels including Philips iDose in the remainder (*n* = 46). Slice thickness was predominantly 5 mm (*n* = 224), with 7 mm (*n* = 35) and 1 mm (*n* = 3) in the remainder; in-plane pixel spacing was 0.6–0.75 mm. Images were resampled to isotropic 1-mm voxels before feature extraction (Supplementary Table S1).

### 2.3 Outcome variables

This study sets 2 types of outcomes as the prediction results of the prediction model. The primary outcome is end-stage renal disease (END-ESRD), defined as long-term dialysis, kidney transplant, or eGFR <15 mL/min/1.73m^2^ for more than three months (15). It takes “whether the patient progresses to ESRD after the first admission due to ANCA” as the outcome variable and verifies the model performance according to ESRD occurred in 1 year, 3 years, and 5 years (END-ESRD-1y; END-ESRD-3y; END-ESRD-5y). The secondary outcome is the renal composite endpoint (END-Composite), which takes “whether the patient has ESRD or all-cause death after the first admission due to ANCA” as the outcome variable, and verifies the model performance according to the composite endpoint occurred in 1 year, 3 years, and 5 years (END-Composite-1y; END-Composite-3y; END-Composite-5y). Meanwhile, the PUMCH-ANCA dataset was stratified into 5 parts according to the renal composite endpoint, resulting in 5 sub-datasets (PUMCH-ANCA-1 to PUMCH-ANCA-5, Supplementary Table S2). The 3-year ESRD outcome was designated as the primary endpoint, given the mean follow-up of ∼3.4 years; the 5-year outcome was analysed as an exploratory endpoint. The exploratory 5-year endpoint was additionally analysed in the restricted cohort of patients with determinable 5-year status (*n* = 156): patients alive at last contact before 60 months and ESRD-free (*n* = 106) were excluded, while patients who reached ESRD within 5 years and those who died within 5 years without ESRD (*n* = 16) were retained. Cox proportional-hazards models (univariable and adjusted for serum creatinine and C3) were fitted with follow-up truncated at 36 and 60 months; the proportional-hazards assumption was assessed using Schoenfeld residuals. Deaths were censored at the date of death in the ESRD-specific analysis.

## 3 Radiomics feature extraction and preprocessing

### 3.1 Renal region segmentation

Renal parenchymal segmentation was automatically performed using the commercial medical image segmentation software Pair (16) (https://www.aipair.com.cn/en/, v3.3.0, AutoSeg module). Segmentation was restricted to the bilateral renal parenchyma, excluding perirenal fat, renal sinus and vascular structures. The automated segmentations of all 262 cases were independently reviewed by two radiologists on two separate occasions, with consistent conclusions regarding segmentation quality. Because the automated segmentation is deterministic (identical input yields an identical segmentation), inter-observer and intra-observer variability are zero by definition, and the intraclass correlation coefficient (ICC) is therefore not applicable.

### 3.2 Radiomics feature extraction

Using the PyRadiomics 3.0.1 toolkit, radiomics features were extracted from the segmented renal region: morphological features reflecting the overall structure of the kidney (including renal volume, long diameter, sphericity, etc.), first-order statistical features reflecting the overall characteristics of image gray distribution (including mean, standard deviation, skewness, kurtosis, etc.), and texture features reflecting the subtle differences in the spatial distribution of image gray [including correlation, contrast, energy based on the gray level co-occurrence matrix (GLCM), long-run emphasis, short-run emphasis based on the gray level run length matrix (GLRLM), etc.].

### 3.3 Feature preprocessing

To mitigate the interference of noise features on model performance, a three-step preprocessing pipeline was implemented on the extracted radiomic features and clinical markers: (1) missing value processing: features with a missing rate exceeding 20% were directly removed; (2) standardization: Continuous variables were normalized using Z-score standardization to eliminate dimensional discrepancies; (3) multicollinearity elimination: Pearson linear correlation coefficients were calculated for all pairwise features. For feature pairs with a correlation coefficient > 0.7, the redundant feature was excluded following discussion and clinical validation by experienced clinicians.

### 3.4 Feature selection method

Feature selection was hypothesis-driven and pre-specified. The imaging predictor (renal volume) was specified *a priori* based on the rationale that renal cortical atrophy reflects irreversible nephron loss and predicts progression to ESRD. The clinical predictors (serum creatinine and complement C3) were likewise pre-specified. No data-driven feature selection was performed. All preprocessing (median imputation and standardization) was performed within each training fold and transferred to the corresponding test fold (Supplementary Table S3).

## 4 Prediction model construction and evaluation

### 4.1 Model construction

Based on the selected training feature pool, two types of prediction models were constructed based on the XGBoost algorithm (learning rate: 0.1, max depth: 6, n_estimators: 100, fixed *a priori* and not tuned on the evaluation folds, a sensitivity analysis across max_depth values confirmed that results were insensitive to this hyperparameter): (a) ESRD prediction model: taking “whether the patient progresses to ESRD within 1 year/3 years/5 years after the first admission due to ANCA” as the outcome variable; (b) Renal composite endpoint prediction model: taking “whether the patient has ESRD or all-cause death within 1/3/5 years after the first admission due to ANCA” as the outcome variable. Training was repeated 5 times for each endpoint prediction. Each time, 4 of the 5 subsets (PUMCH-ANCA-1 to PUMCH-ANCA-5) were used as the training set, and the remaining 1 was used as the test set. During model evaluation and interpretability analysis, comprehensive evaluation was carried out based on the results of 5 trainings to avoid performance deviation caused by dataset division to the greatest extent.

### 4.2 Model evaluation indicators

A multi-dimensional indicator set was used:

ROC-AUC: quantifies the model's ability to distinguish between event occurrence and non-occurrence.
Six-dimensional radar chart: visualizes accuracy (ACC), specificity (SPE), sensitivity (SEN), positive predictive value (PPV), negative predictive value (NPV), and F1 score.
Decision curve analysis (DCA): calculates the net benefit of model-guided intervention at different risk thresholds, compared with “intervene for all” and “intervene for none” strategies.
Calibration curve: plots the average predicted probability of each group against the observed event frequency to assess prediction reliability.

### 4.3 Model interpretability analysis

SHAP (SHapley Additive exPlanations) values were used to interpret the model. SHAP values of each feature in the test subsets were calculated (larger absolute value indicates higher contribution). SHAP summary plots were used to show the distribution of SHAP values and the direction of influence of core features (e.g., renal volume, creatinine) on outcome events.

### 4.4 KM risk stratification

Based on the training feature pool obtained in 2.4, to prevent data leakage, patients were stratified by the median of each pre-specified indicator; the Kaplan–Meier curves serve as a descriptive illustration, and the primary inference derives from the pre-specified regression models, patients were then divided into high-risk and low-risk subgroups. Kaplan–Meier survival analysis was then performed for each indicator, and survival curves were plotted over time (months). Bonferroni correction was applied to control type I error from multiple hypothesis testing, and the Log-rank test was used to calculate the adjusted *P* value for survival differences between subgroups.

## 5 Results

This study recruited 262 participants. 49% of participants were male (*n* = 128) and the mean age is 57.4 ± 15.9 years. The mean sCr level for all participants is 252.5 ± 222.5 μmol/L and the overall mean volume of the (left and right) kidney is 135.7 mL ±47.4 mL (Table 1). The mean follow-up duration was 3.4 years. Ultimately, 23% (59 out of 262) of patients developed ESRD, and 11% (29 out of 262) of patients reached the endpoint of death (Table 2). The distribution of ESRD and mortality cases across subsets was comparable to the overall distribution.

**Table 1:** Clinical characteristics of patients in PUMCH-ANCA dataset.

Clinical Characteristics	Total *N* = 262	PUMCH-ANCA-1 *N* = 53	PUMCH-ANCA-2 *N* = 53	PUMCH-ANCA-3 *N* = 52	PUMCH-ANCA-4 *N* = 52	PUMCH-ANCA-5 *N* = 52
Age, mean(SD)	57.4 (15.9)	53.8 (15.9)	53.6 (16.5)	61.3 (14.8)	58.5 (17.3)	59.7 (13.6)
Male sex, *n* (%)	128 (49)	21 (40)	28 (53)	26 (50)	30 (58)	23 (44)
sCr (*μ*mol/L), mean(SD)	252.5 (222.5)	241.1 (207.0)	261.5 (232.9)	255.5 (232.2)	229.4 (192.1)	275.2 (249.3)
eGFR(mL/min/1.73m^2^), mean(SD)	49.8 (42.7)	49.5 (42.2)	53.2 (47.1)	48.2 (40.8)	52.8( 43.1)	45.3 (41.1)
Mean kidney Volume(mL), mean(SD)	135.7 (47.4)	121.2 (36.9)	138.7 (52.7)	134.8 (50.3)	144.7 (46.5)	139.4 (47.2)

**Table 2:** Distribution of follow-up durations and outcomes of patients in PUMCH-ANCA dataset.

Cohorts	Total *N* = 262	PUMCH-ANCA-1 *N* = 53	PUMCH-ANCA-2 *N* = 53	PUMCH-ANCA-3 *N* = 52	PUMCH-ANCA-4 *N* = 52	PUMCH-ANCA-5 *N* = 52
Follow-up, yr	3.4 (3.2)	3.4 (3.1)	3.5 (3.2)	3.5 (3.2)	3.2 (3.2)	3.4 (3.4)
ESRD, n(%)	59 (23)	13 (25)	13 (25)	11 (21)	11 (21)	11 (21)
Death, n(%)	29 (11)	4 (8)	4 (8)	8 (15)	6 (12)	7 (13)
Death without ESRD, n(%)	20 (8)	3 (6)	3 (6)	5 (10)	4 (8)	5 (10)

### 5.1 ESRD prediction model performance

We conducted a multidimensional evaluation of the ANCA-associated vasculitis predictive model for predicting ESRD. The combined model (renal volume + sCr + C3) was compared with the clinical-only model (sCr + C3). For the primary 3-year ESRD endpoint (*n* = 262, 56 events), renal volume was independently associated with ESRD (Wald *p* = 0.004, OR per SD = 0.465, 95% CI 0.277–0.782; likelihood-ratio test *p* = 0.002), improved risk reclassification (IDI =  + 0.041, bootstrap *p* = 0.024; category-free NRI =  + 0.817, bootstrap *p* = 0.012), and improved the Brier score (0.084 → 0.074), with calibration preserved (intercept −0.045 → −0.029; slope 0.96 → 0.95). The AUC increased from 0.91 to 0.93 (DeLong *p* = 0.20 for this nested comparison; Figures 3E–H). The radiomics-only model showed limited standalone discrimination (AUC ≈ 0.7). The association was not confounded by CT acquisition factors: after additional adjustment for scanner vendor and slice thickness, the odds ratio for renal volume was 0.475 (95% CI 0.277–0.814, *p* = 0.007), with direction-consistent results across vendor and slice-thickness subgroups (Supplementary Table S4). For the exploratory 1-year endpoint (*n* = 262, 46 events), the clinical and combined models achieved AUCs of 0.92 and 0.93, respectively (Figures 3A–D). In the restricted 5-year cohort (*n* = 156, 59 events), renal volume remained independently associated with ESRD (Wald *p* = 0.034, OR per SD 0.528, 95% CI 0.293–0.952; likelihood-ratio test *p* = 0.029; IDI +0.028, bootstrap *p* = 0.048), with preserved discrimination (AUC ≈ 0.91–0.92) and negligible incremental AUC (+0.004). In Cox analysis, renal volume was strongly associated with ESRD (HR per SD 0.573, 95% CI 0.420–0.781, *p* < 0.001), with a direction-consistent attenuated estimate after adjustment for sCr and C3 (3-year HR 0.878, 95% CI 0.677–1.139, *p* = 0.33; 5-year HR 0.872, 95% CI 0.675–1.12 6, *p* = 0.29), reflecting the prognostic information shared between renal volume and these clinical markers; the proportional-hazards assumption was not materially violated (*p* = 0.068), and landmark analyses were consistent with a post-first-year effect.

**Figure 3:**
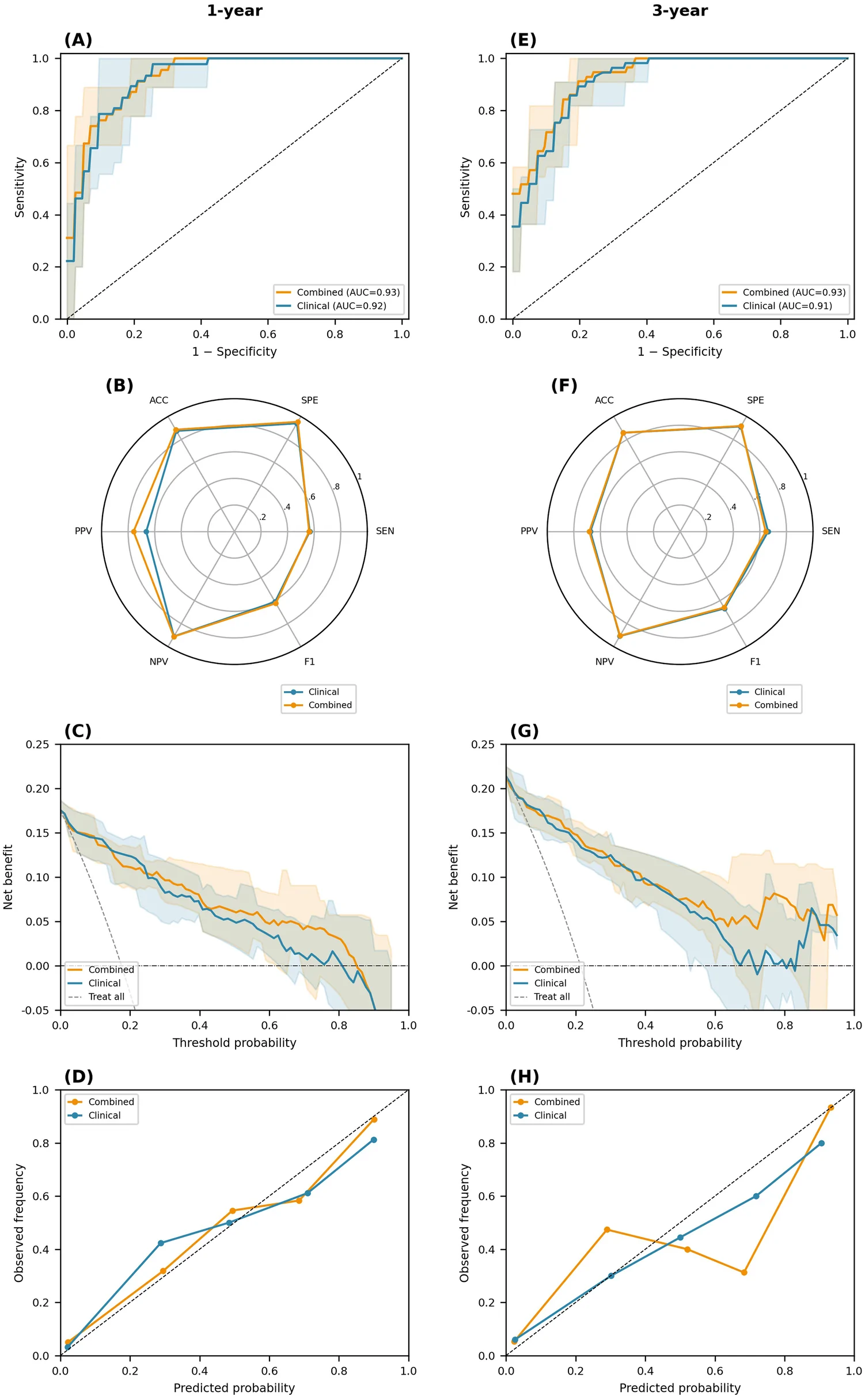
Predictive performance of the clinical-only model (sCr + C3) and the combined model (renal volume + sCr + C3) for ESRD. ROC curves **(A,E)**, radar charts of model performance (SEN, SPE, ACC, PPV, NPV and F1 score at a fixed threshold of 0.5; B, F), decision-curve analysis **(C,G)** and calibration curves **(D,H)** for the exploratory 1-year endpoint (A–D, left column) and the primary 3-year endpoint (**E–H**, right column). AUCs were computed from pooled out-of-fold predictions of stratified five-fold cross-validation (1-year: clinical 0.92, combined 0.93; 3-year: clinical 0.91, combined 0.93; DeLong *p* = 0.20 for the 3-year nested comparison). Shaded bands represent 95% confidence intervals across folds (ROC) or across bootstrap resamples (decision curve). For decision-curve analysis, the grey lines represent the strategies of treating all and treating none; a higher net benefit suggests potential clinical utility within the threshold ranges shown. The 1-year analysis is exploratory; 1-year status was determinable for 235/262 (90%) of patients. ESRD, end-stage renal disease; ROC, receiver operating characteristic; AUC, the area under the ROC curve; SEN, sensitivity; SPE, specificity; ACC, accuracy; PPV, positive predictive value; NPV, negative predictive value; DCA, decision curve analysis.

### 5.2 Renal composite endpoint prediction model performance

For the secondary 3-year composite outcome (67 events), renal volume was independently associated with the composite outcome (Wald *p* = 0.025, OR per SD 0.630, 95% CI 0.421–0.943; likelihood-ratio test *p* = 0.020), improved risk reclassification (IDI =  + 0.019, bootstrap *p* = 0.018; category-free NRI =  + 0.483, bootstrap *p* = 0.024), improved the Brier score (0.138–0.131). The combined model improved discrimination (AUC 0.81–0.84, pooled out-of-fold; *Δ*AUC =  + 0.026, DeLong *p* = 0.05; Figures 4E–H). Radar-chart performance metrics across the validation folds are shown in Figures 4B–F (fixed pre-specified decision threshold of 0.5), and decision-curve analysis suggested potential clinical utility within the threshold ranges shown (Figures 4C–G). For the exploratory 1-year endpoint, the clinical and combined models achieved AUCs of 0.82 and 0.85, respectively (Figures 4A–D).

**Figure 4:**
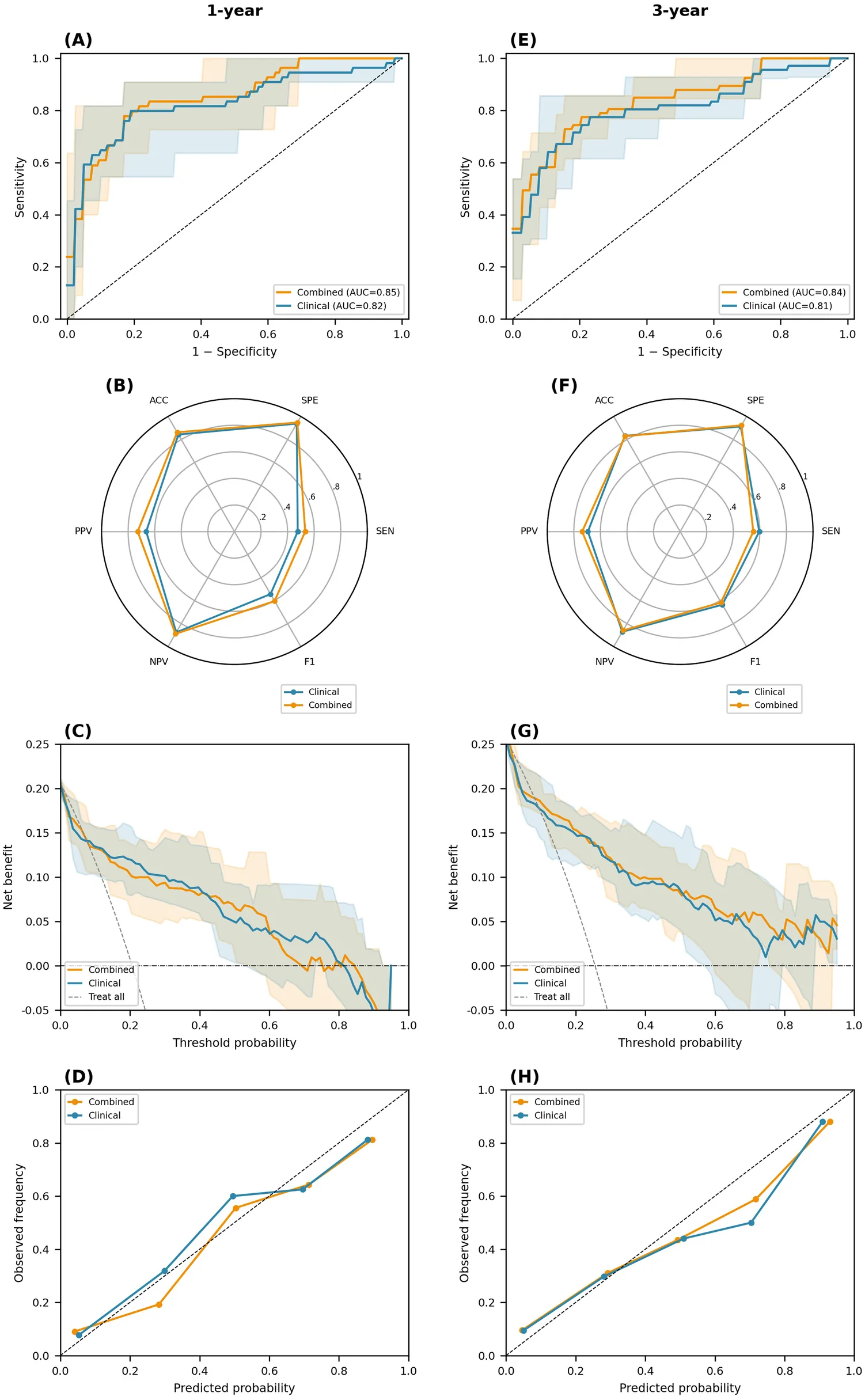
Predictive performance of the clinical-only model (sCr + C3) and the combined model (renal volume + sCr + C3) for the renal composite outcome (incident ESRD or all-cause death). ROC curves **(A,E)**, radar charts **(B,F)**, decision-curve analysis **(C,G)** and calibration curves **(D,H)** for the exploratory 1-year endpoint **(A–D)** and the 3-year endpoint **(E–H)**. AUCs were computed from pooled out-of-fold predictions of stratified five-fold cross-validation (1-year: clinical 0.82, combined 0.85; 3-year: clinical 0.81, combined 0.84; *Δ*AUC =  + 0.026, DeLong *p* = 0.05 for the 3-year comparison). Shaded bands and strategy lines as in Figure 3. The combined model includes the three pre-specified predictors (renal volume, serum creatinine and C3). ESRD, end-stage renal disease; ROC, receiver operating characteristic; AUC, the area under the ROC curve; SEN, Sensitivity; SPE, specificity; ACC, accuracy; PPV, positive predictive value; NPV, negative predictive value; DCA, decision curve analysis.

### 5.3 Model interpretability analysis

SHAP analysis of the combined model (renal volume + sCr + C3) demonstrated that, for the primary 3-year ESRD endpoint, serum creatinine at admission was the dominant contributor to model predictions, followed by C3, with renal volume contributing a smaller but consistent share (Figuress 5A,B); higher predicted ESRD risk was associated with higher serum creatinine, lower C3, and smaller renal volume. The composite-outcome model showed the same ordering of feature contributions (Figures 5C,D).

**Figure 5:**
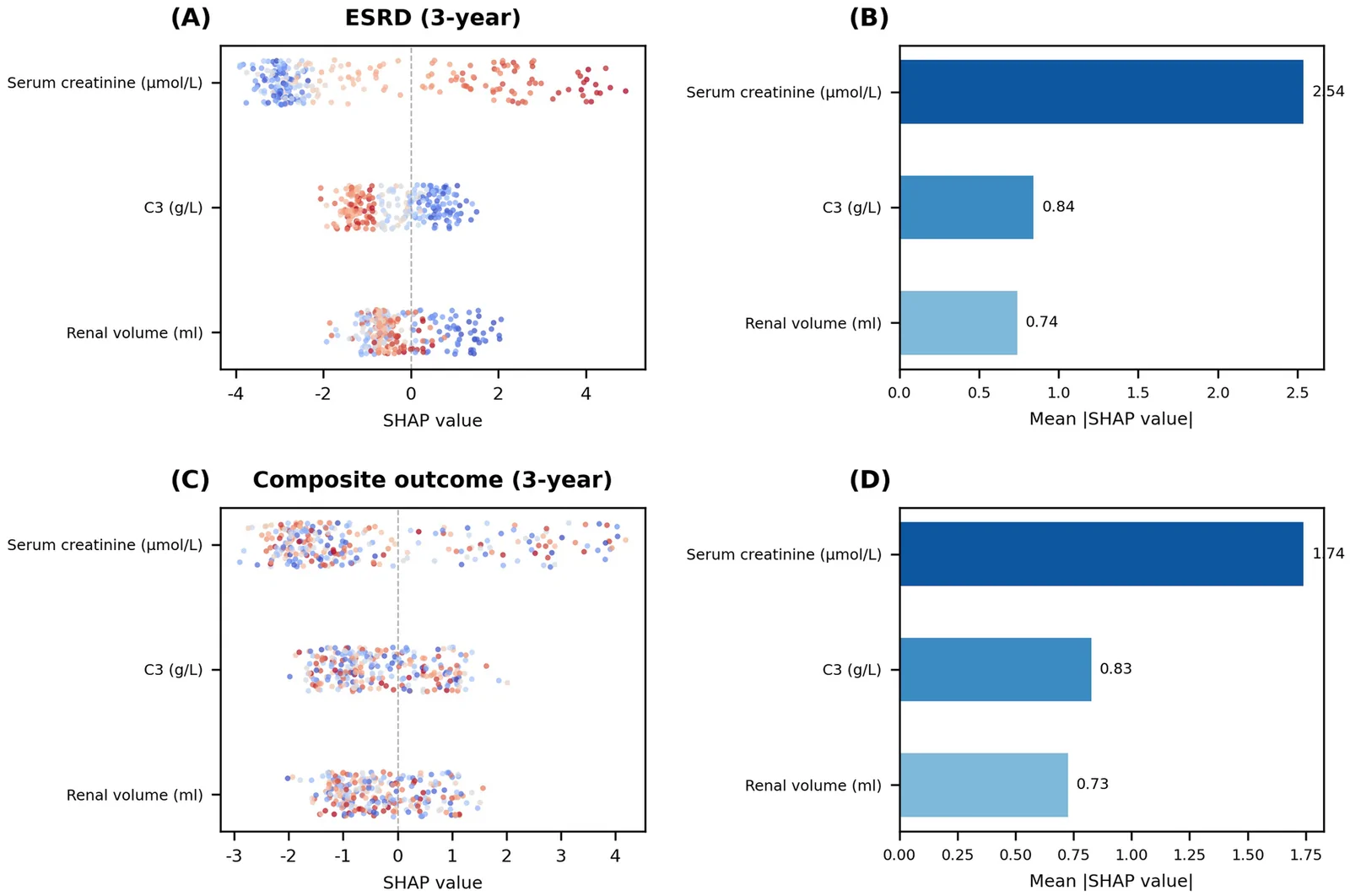
SHAP analysis of the combined model (renal volume + sCr + C3). SHAP summary plots **(A,C)** and mean absolute SHAP values **(B,D)** for the primary 3-year ESRD endpoint **(A,B)** and the 3-year renal composite outcome (incident ESRD or all-cause death; C, D). Each dot in the summary plots represents one patient; the *x*-axis shows the SHAP value, i.e., the impact of the feature on the model output (right: higher predicted risk; left: lower risk); the colour indicates the within-cohort rank of the feature value (blue = lower, red = higher). Higher serum creatinine, lower C3 and smaller renal volume shift predictions toward the respective outcome. Serum creatinine was the dominant contributor, followed by C3, with renal volume contributing a smaller but consistent share, and the composite-outcome model showed the same ordering. SHAP values were computed for each patient from out-of-fold predictions of the nested cross-validation framework.

### 5.4 KM survival curve analysis

Kaplan–Meier survival analysis demonstrated that, for 3-year ESRD (Figures 6A–C), patients with renal volume > 130.6 mL, serum creatinine ≤ 177.5 μmol/L, or C3 > 0.97 g/L had significantly higher ESRD-free survival (log-rank *p* = 0.002, *p* < 0.001, and *p* < 0.001, respectively). Cut-offs were pre-specified as the cohort medians, independently of outcome data (Supplementary Table S5); the curves serve as a descriptive illustration of these univariate associations, whereas the primary inference derives from the pre-specified regression models.

**Figure 6:**
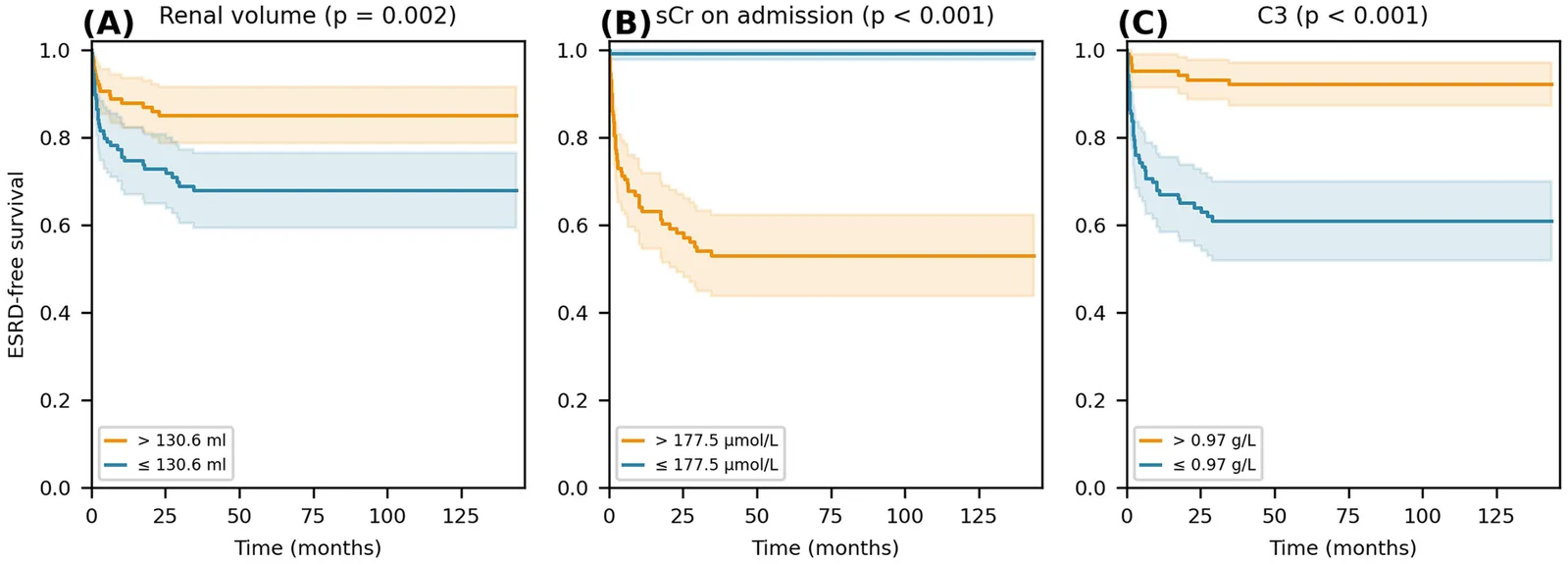
Kaplan–meier curves for 3-year ESRD-free survival according to pre-specified median-based stratification. **(A)** Renal volume (> 130.6 mL vs. ≤ 130.6 mL; log-rank *p* = 0.002), **(B)** serum creatinine (> 177.5 μmol/L vs. ≤ 177.5 μmol/L; log-rank *p* < 0.001) and **(C)** complement C3 (> 0.97 g/L vs. ≤ 0.97 g/L; log-rank *p* < 0.001). Shaded bands represent 95% confidence intervals. Cut-offs were pre-specified as cohort medians, independently of outcome data; the curves serve as a descriptive illustration of the univariate associations, whereas the primary inference derives from the pre-specified regression models (see Methods). ESRD, end-stage renal disease; sCr, serum creatinine; C3, complement component 3.

## 6 Discussion

This study represents the first large-cohort investigation to integrate clinical parameters with renal CT radiomics for the non-invasive prediction of long-term renal outcomes in AAV. Our most significant innovation lies in the development of a multi-timepoint prognostic model that effectively combines routinely available biomarkers—specifically, sCr and C3 at presentation—with a key macroscopic structural feature derived from radiomics, namely renal volume. The model demonstrated high predictive accuracy, particularly for the primary 3-year ESRD endpoint (AUC 0.93 vs 0.91 for the clinical-only model), with renal volume independently associated with ESRD and significantly improving risk reclassification. This robust performance not only validates the prognostic relevance of the identified parameters but also underscores the complementary value of integrating clinical and imaging data to surpass the predictive power of either modality alone.

Our model converges with and extends existing AAV prognostic tools. We compared our model with the ANCA Renal Risk Score (ARRS) (5), the improved ANCA Kidney Risk Score (AKRiS) (8), and the Percentage of ANCA Crescentic Score (PACS) (17). In the 52 patients of our cohort in whom these biopsy-based scores could be computed, our non-invasive model achieved an AUC of 0.85 for 3-year ESRD, comparable to the AKRiS (0.84) and higher than the ARRS (0.76), while remaining entirely non-invasive; PACS could not be computed because crescent quantification was not available in our retrospective dataset. Both scores are biopsy-dependent, whereas our model offers complementary value when biopsy is not feasible or not repeatable for serial monitoring (17). This provides comparable prognostic insight with greater clinical accessibility and patient safety. The core value of radiomics lies in accurately extracting renal volume via CT-based 3D quantification, objectively capturing cumulative parenchymal loss from recurrent inflammation and small-vessel injury in AAV. It forms a “structure-function-inflammation” triad with sCr and C3, significantly enhancing model accuracy. To our knowledge, this is the first systematic construction and validation of a multi-timepoint radiomics-clinical model specifically for AAV, beyond prior studies in CKD (18, 19) or diabetic nephropathy (20).

Regarding all-cause mortality, our composite endpoint (ESRD or death) captures well-established risk factors including MPA subtype, advanced age, high-titer PR3-ANCA, BVAS, pulmonary involvement/infection, and anemia (21–25), and failure to recover renal function (26). By incorporating age, sCr, and radiomics-derived renal volume, our model enhances risk stratification with a novel dimension of renal structural integrity, offering a more holistic assessment than models based solely on clinical or pathological data.

Our study further identified complement C3 as a significant predictor for both renal and composite endpoints, with its decreased serum level being associated with poorer outcomes. This observation aligns with a growing body of evidence highlighting the critical role of complement activation in AAV. Specifically, a reduced C3 level is more frequently observed in patients presenting with acute kidney injury (AKI) and serves as an independent risk factor for progressive renal disease (27, 28). The pathophysiological link can be attributed to the alternative complement pathway, which is now recognized as a central driver in AAV pathogenesis (29–31). In this pathway, spontaneous hydrolysis of C3 initiates the formation of C3 convertase (C3bBb), which cleaves additional C3 molecules into C3a and C3b. The deposition of C3b on target surfaces, such as activated neutrophils and endothelial cells, creates a powerful amplification loop, leading to massive complement cascade activation, membrane attack complex (C5b-9) formation, and subsequent neutrophil activation and tissue injury. Thus, the consumption of C3 reflected by its low serum level may indicate heightened systemic complement activation, which correlates with more severe renal damage and a worse prognosis, as evidenced in our cohort. The inclusion of C3 in our model effectively captures this key pathogenic process, enhancing the biological plausibility and prognostic accuracy of our prediction tool.

An important observation from our study is that the predictive performance of the composite endpoint model remained comparable across the 1-, 3-, and 5-year outcomes, with AUCs of 0.85, 0.84, and 0.82 for the combined model, respectively. The clinical course of AAV differs markedly across disease phases. The initial year following diagnosis is typically dedicated to intensive immunosuppressive induction therapy, a period characterized by heightened disease activity, severe clinical presentations, and an elevated frequency of treatment-related complications, particularly infections. Indeed, prior studies have consistently identified infection as the leading cause of death during the first year in AAV patients (21). Furthermore, as AAV predominantly affects an older population, long-term outcomes are increasingly influenced by age-related comorbidities and non-vasculitic causes of death over time. The fact that our model, which was trained on long-term composite endpoints, accurately reflects this shift in dominant risk factors from early treatment-related complications to later, age-related events, strengthens its validity and clinical relevance. This dynamic performance underscores the model's ability to encapsulate the evolving nature of mortality risk throughout the disease continuum.

The prediction model constructed in this study has important clinical application value in three aspects. First, it provides a non-invasive alternative for patients who cannot undergo renal biopsy, and clinicians can quickly assess the renal prognosis risk by combining routine CT examination and basic laboratory indicators, avoiding the complications caused by invasive operations. Second, the multi-time point prediction function (1/3/5 years) can help clinicians formulate personalized follow-up and treatment plans: for high-risk patients identified by the model, more intensive immunosuppressive therapy and more frequent renal function monitoring can be adopted; for low-risk patients, the treatment intensity can be appropriately reduced to avoid over-treatment-related side effects. Third, the core features identified in the study (renal volume, sCr, C3) are easy to obtain in clinical practice, and the model has good operability, which is expected to be integrated into the clinical decision support system to assist in real-time prognosis assessment.

This study has several limitations that need to be noted. First, it is a single-center retrospective cohort study, and the sample size (262 cases) is relatively limited. The results were derived from internal cross-validation only, without an independent external validation cohort. The selection bias may exist, and the model's external validity needs to be verified by multi-center prospective studies. Second, the study did not include other potential influencing factors such as treatment response and genetic markers, which may further improve the model's predictive performance if added. Third, the follow-up duration of some patients was shorter than five years, making their outcome status in the exploratory 5-year analyses indeterminate; sensitivity analyses restricted to patients with determinable 5-year status yielded consistent results, and competing-risk models were not applied. In addition, patients with failed or unreliable segmentation were excluded, and results may not fully generalize to this subgroup.

## 7 Conclusion

The renal prognosis prediction model for AAV patients based on “renal CT radiomics features + clinical markers” has high predictive accuracy and good clinical operability. It can effectively identify patients at high risk of ESRD and renal composite endpoints at multiple time points and provides a new non-invasive strategy for clinical precise risk stratification and individualized treatment. Future studies should expand multi-center prospective cohorts, optimize feature selection, and further improve the model's generalization and stability to promote its clinical transformation and application.

## Data Availability

The datasets presented in this article are not readily available because all raw clinical records and CT images are confidential medical data of Peking Union Medical College Hospital. Fully de-identified clinical, radiomic and follow-up data cannot be shared, released or reused for other research without ethics and hospital approval. Raw imaging and individual-level records will not be deposited in public repositories to comply with patient privacy regulations. This dataset is only permitted for the present AAV renal prognosis modeling study. Requests to access the datasets should be directed to Zuoxian Hou, houzuoxian@pumch.cn.

## Supplementary material

Supplementary Table S1. CT acquisition protocol.

Supplementary Table S2. Stratified five-fold cross-validation breakdown.

Supplementary Table S3. Feature-reduction flow.

Supplementary Table S4. Sensitivity analysis of renal volume by scanner manufacturer and slice thickness (3-year ESRD).

Supplementary Table S5. Pre-specified median cut-offs for Kaplan–Meier stratification (3-year ESRD).
