# Supplementary material for: Radiomics-Based prediction of renal prognosis in ANCA-associated vasculitis
Source: Front Digit Health. 2026 Sep 30;8:1915391. doi: 10.3389/fdgth.2026.1915391 (PMC13662048; doi:10.3389/fdgth.2026.1915391)
Supplement: Supplementary file 1 [file Table1.docx]

# Supplementary Tables

## Table S1. CT acquisition protocol

| Parameter | Distribution |
| --- | --- |
| Scanner vendor | Siemens (n = 213), Philips (n = 34), GE (n = 15) |
| Contrast | Non-contrast |
| Tube voltage | 120 kV (n = 208), 140 kV (n = 35), 100 kV (n = 15), 110 kV (n = 3), 90 kV (n = 1) |
| Reconstruction kernel | B30f (n = 168), B25f (n = 18), B31f (n = 15), STANDARD/GE (n = 15), other kernels incl. Philips iDose (n = 46) |
| Slice thickness | 5 mm (n = 224), 7 mm (n = 35), 1 mm (n = 3) |
| In-plane pixel spacing | 0.6–0.75 mm |
| Resampling | Isotropic 1-mm voxels |

Note: reconstruction-kernel and tube-voltage values were read from the DICOM headers of the series actually used for feature extraction (series-level match on the series description); all values sum to the full cohort (n = 262).

## Table S2. Stratified five-fold cross-validation breakdown

| Fold | Training (n) | Test (n) | 3-year ESRD events | 3-year composite events |
| --- | --- | --- | --- | --- |
| 1 | 209 | 53 | 12 | 14 |
| 2 | 209 | 53 | 11 | 14 |
| 3 | 210 | 52 | 11 | 13 |
| 4 | 210 | 52 | 11 | 13 |
| 5 | 210 | 52 | 11 | 13 |
| Total | — | 262 | 56 | 67 |

## Table S3. Feature-reduction flow

| Stage | Description | n |
| --- | --- | --- |
| Radiomic features extracted | Shape, first-order, and texture features | 107 |
| Redundant or derived radiomic features removed | Duplicate size and diameter measurements, etc. | 17 |
| Radiomic features retained | — | 90 |
| Clinical variables excluded (outcome-related or post-baseline) | Dialysis status, discharge creatinine, eGFR | 3 |
| Clinical variables excluded (>20% missing) | — | 9 |
| Final pre-specified predictors | Renal volume, serum creatinine, complement C3 | 3 |

## Table S4. Sensitivity analysis of renal volume by scanner manufacturer and slice thickness (3-year ESRD)

| Subgroup | n (events) | Wald P | OR per SD (95% CI) |
| --- | --- | --- | --- |
| Overall (sCr + C3) | 262 (56) | 0.004 | 0.465 (0.277–0.782) |
| + manufacturer + slice thickness | 262 (56) | 0.007 | 0.475 (0.277–0.814) |
| Siemens | 213 (46) | 0.010 | 0.445 (0.241–0.821) |
| Philips | 34 (7) | 0.59 | 0.743 (0.255–2.163) |
| GE | 15 (3) | — | — |
| 5-mm slice thickness | 224 (46) | 0.002 | 0.357 (0.187–0.684) |
| 7-mm slice thickness | 35 (9) | 0.08 | 0.087 (0.006–1.332) |

## Table S5. Pre-specified median cut-offs for Kaplan–Meier stratification (3-year ESRD)

| Variable | Median cut-off | Log-rank P |
| --- | --- | --- |
| Renal volume | > 130.6 ml | 0.002 |
| Serum creatinine | > 177.5 μmol/L | < 0.001 |
| Complement C3 | > 0.97 g/L | < 0.001 |
